# Consistency analysis and conversion model establishment of mini-mental state examination and montreal cognitive assessment in Chinese patients with Alzheimer’s disease

**DOI:** 10.3389/fpsyg.2022.990666

**Published:** 2022-09-23

**Authors:** Lu Zhou, Zhichuan Lin, Bin Jiao, Xinxin Liao, Yafang Zhou, Hui Li, Lu Shen, Ling Weng

**Affiliations:** ^1^Department of Neurology, Xiangya Hospital, Central South University, Changsha, Hunan, China; ^2^Department of Neurology, Hainan General Hospital, Haikou, Hainan, China; ^3^National Clinical Research Center for Geriatric Disorders, Central South University, Changsha, China; ^4^Engineering Research Center of Hunan Province in Cognitive Impairment Disorders, Central South University, Changsha, China; ^5^Hunan International Scientific and Technological Cooperation Base of Neurodegenerative and Neurogenetic Diseases, Changsha, China; ^6^Key Laboratory of Hunan Province in Neurodegenerative Disorders, Central South University, Changsha, China; ^7^Department of Geriatrics, Xiangya Hospital, Central South University, Changsha, Hunan, China; ^8^Key Laboratory of Organ Injury, Aging and Regenerative Medicine of Hunan Province, Changsha, China

**Keywords:** MMSE-C, MoCA-BJ, AD, circle-arc method, consistency analysis, conversion model

## Abstract

**Background:**

The Chinese version of the Mini-Mental State Examination (MMSE-C) and the Beijing version of the Montreal Cognitive Assessment (MoCA-BJ) are the most commonly used scales to screen for Alzheimer’s disease (AD) among Chinese patients; however, their consistency varies according to populations and languages. Equivalent conversion of MMSE-C and MoCA-BJ scores is important for meta-analysis.

**Materials and methods:**

MMSE-C and MoCA-BJ scoring were performed on the enrolled patients with AD (*n* = 332). Consistency analysis of MMSE-C and MoCA-BJ scores of patients in the conversion groups was performed. The circle-arc method was used to convert the MMSE-C scores of the conversion groups into MoCA-BJ scores, and the conversion formula was generated. The MMSE-C data of the verification group was converted to MoCA-BJ according to the formula, and the consistency analysis of the original MoCA-BJ of the verification group and the converted MoCA-BJ was performed to verify the conversion model.

**Results:**

The results of the consistency analysis of MMSE-C and MoCA-BJ in group A showed that the correlation coefficients of the total group, high education years subgroup, medium education years subgroup, and low education years subgroup were 0.905 (*P* < 0.001), 0.874 (*P* < 0.001), 0.949 (*P* < 0.001), and 0.874 (*P* < 0.001), respectively, with high consistency and statistical significance. After applying the circle-arc method for equivalent conversion, the consistency analysis results of the original and the converted MoCA-BJ of the patients in group B of the total group, high education years subgroup, medium education years subgroup, and low education years subgroup were 0.891 (*P* < 0.001), 0.894 (*P* < 0.001), 0.781 (*P* < 0.001), 0.909 (*P* < 0.001), respectively, with high consistency and statistical significance.

**Conclusion:**

We established and validated a model of MMSE-C and MoCA-BJ score conversion for Chinese patients with AD using the circle-arc method. This model could be useful for multi-centers clinical trials and meta-analysis.

## Introduction

Alzheimer’s disease (AD) is a neurodegenerative disease characterized by progressive cognitive dysfunction and abnormal mental behavior (Sperling et al., 2011). Its two diagnostic neuropathological hallmarks are numerous extracellular deposits of amyloid-β (Aβ) plaques and neurofibrillary tangles (Braak and Braak, 1996). Neuropsychological assessment is used to assist in the diagnosis of AD and evaluate the efficacy of the treatment (Ashford et al., 2006). Currently, there are various neurological assessment scales in clinical practice (Tsoi et al., 2015); the Mini-Mental State Examination (MMSE) (Folstein et al., 1975) and the Montreal Cognitive Assessment (MoCA) (Nasreddine et al., 2005) are the two most common ones.

The MMSE scale covers six aspects, including orientation (time and place), registration, attention and calculation, recall, language (naming, retelling, listening and understanding, reading, and writing), and visual construction, with a total score of 30 (Folstein et al., 1975). The MMSE score abnormal interpretation criteria varies according to the educational levels of the subjects (Tombaugh and McIntyre, 1992).

The MoCA scale involves eight cognitive domains, including attention and concentration, executive function, memory, language, visuospatial skills, abstract thinking, computation, and orientation, with a total score of 30 (Nasreddine et al., 2005). The boundary value of the MoCA scale is 26 points for normal and cognitive impairment. If the duration of education of the subject is not more than 12°years, one point is added to the original score (Lee et al., 2008; Siciliano et al., 2019).

The MMSE is greatly affected by age, socioeconomic status, and education level (Crum et al., 1993), for example, it is not sensitive enough for elderly subjects (> 75–80°years), patients with mild cognitive impairment, and subjects with a high education level (Nys et al., 2005). Meanwhile, MoCA showed a higher detection rate for mild cognitive impairment (MCI) than MMSE and a lower detection rate for moderate and severe dementia than MMSE (Roalf et al., 2012; Huang et al., 2018). MoCA includes more heavily weighted visuospatial and executive function measurements, which may reduce the impact of the ceiling and learning effects (Kasten et al., 2010) but increase the likelihood of the floor effect (Federico et al., 2018). MoCA also has limitations, its data should stratified by age, education, ethnically diverse, and population (Dong et al., 2013). MoCA is less widely used as MMSE (Dong et al., 2013).

Mini-mental state examination (MMSE) and MoCA can be affected by differences in culture and language (Larner, 2012; Verghese et al., 2012). The MMSE and MoCA have been translated into local Chinese language. In China, the Chinese version of the MMSE (MMSE-C) (Zhang, 2003) and the Beijing version of the Montreal Cognitive Assessment (MoCA-BJ) (Yu et al., 2012) are the two most widely used.

Given that both scales are widely used in clinical screening, as well as in clinical trials and cohort studies, a rule to facilitate conversions and comparison of data from different centers and clinical trials would be essential (Wong et al., 2013; Helmi et al., 2016). Therefore, the establishment of validity consistency conversion of MMSE-C and MoCA-BJ is not only conducive to the continuity of cognitive tracking in clinical settings but also to the comparison and integration of cognitive data from heterogeneous longitudinal studies.

Therefore, this study had the following aims: (1) to estimate the level of agreement between MMSE-C and MoCA-BJ within the total and different educational levels Chinese AD patients, (2) to derive a conversion model for the two scales using the circle-arc method, and (3) to validate the conversion model in a small sample of patients with AD.

## Materials and methods

### Participants

We enrolled 322 patients who met the diagnostic criteria of “clinically possible and probable AD” in the 2011 NIA-AA diagnostic guidelines, from January 1, 2016, to April 1, 2019, at Xiangya Hospital of Central South University. Patients with structural brain lesions (tumor or stroke), and patients with a previous diagnosis of schizophrenia or bipolar disorder with psychotic features were excluded. We included patients who undertook both the MMSE-C and MoCA-BJ in the same session. The study was approved by the Ethics Committee of Xiangya Hospital of Central South University, and the subjects provided informed consent.

### Procedures

Patients with AD (*n* = 322) were randomly divided into the conversion group A (*n* = 161) and the validation group B (*n* = 161) and stratified according to their length of education. The consistency of MMSE-C and MoCA-BJ was analyzed for group A data, and the conversion formula was generated using the Circular-arc method to obtain the MoCA-BJ score from 0 to 30 MMSE-C points. According to the conversion table obtained above, the converted MoCA-BJ was obtained for group B, and the consistency of the formula was verified by comparing the converted MoCA-BJ with the original MoCA-BJ in group B.

### Equivalent conversion method

**FIGURE 1:**
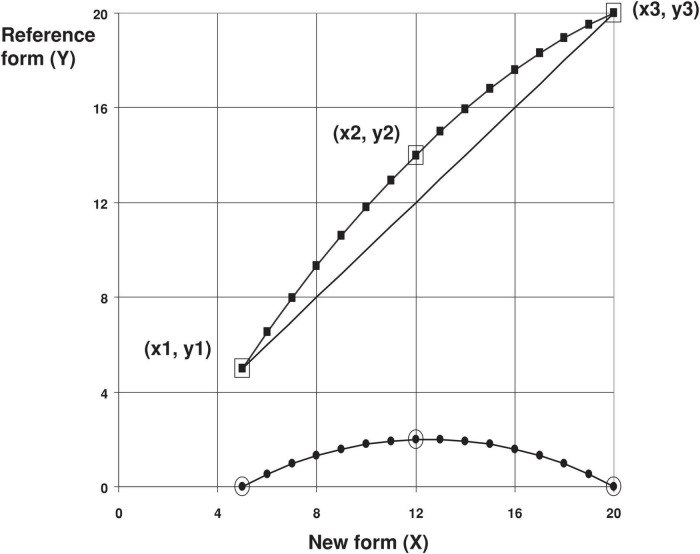
Circle arc method of converted equating.

The circle-arc method (Livingston and Kim, 2009) is an equivalent conversion method for grades and rating scales, and the MoCA-BJ conversion of MMSE-C is more consistent than the linear method. Therefore, this study applies this method to the MoCA-BJ conversion of MMSE-C. According to the nadir (x1, y1), zenith (x3, y3), and midpoint (x2, y2) (Figure 1) of a set of data, the method applies the following formula for calculation and conversion to obtain the converted data.


$$\text{L}\left(x\right)=y_{1}+\frac{{\text{y}}_{3}-{\text{y}}_{1}}{{\text{x}}_{3}-{\text{x}}_{1}}\left(\text{x}-{\text{x}}_{1}\right)$$

$${\text{y}}^{*}=\text{y}-\text{L}\left(x\right)$$

$${\left(\text{X}-{\text{x}}_{c}\right)}^{2}+{\left(\text{Y}-{\text{y}}_{c}\right)}^{2}={\text{r}}^{2}$$

${\text{Y}}^{*}={\text{y}}_{c}+\sqrt{{\text{r}}^{2}-{\left(\text{X}-{\text{x}}_{c}\right)}^{2}}$ (This formula is used when Y* is positive)

${\text{Y}}^{*}={\text{y}}_{c}-\sqrt{{\text{r}}^{2}-{\left(\text{X}-{\text{x}}_{c}\right)}^{2}}$ (This formula is used when Y* is negative)


xc=(x32-x12)2(x3-x1)

yc=(x12)(x3-x2)-(x22+(y2*)2)(x3-x1)+(x32)(x2-x1)2[y2*(x1-x3)]

$$\text{r}=\sqrt{{\left({\text{x}}_{c}-{\text{x}}_{1}\right)}^{2}+{\left({\text{y}}_{c}\right)}^{2}}$$

### Statistical analysis

SPSS 19.0 software was used for data analysis. Categorical variables are expressed in quantity (percentage) and analyzed using the chi-square test. Continuous variables are expressed as mean ± standard deviation or median (interquartile interval, IQR), according to the situation, and analyzed by a *t*-test or Mann–Whitney *U* test. Pearson or Spearman correlation analysis was used to test the consistency, and statistical significance was set at *P* < 0.05.

## Results

### Patients’ characteristics

A total of 322 patients with AD were included in this study. Group A of the total group included 161 patients, their mean age was 69.69 ± 11.12°years, and 68 (42.2%) of them were men. The mean MMSE-C and MoCA-BJ scores were 12.11 ± 6.82 (range, 2–28) and 8.2 ± 5.34 (range, 0–25), respectively. Group B of the total group included 161 patients; the mean age was 70.01 ± 11.21°years and 55 (34.2%) of them were men. The mean MMSE-C and MoCA-BJ scores were 12.27 ± 6.91 (range, 1–27) and 7.91 ± 5.30 (range, 0–22), respectively. Depending on the level of education, the participants were classified into three subgroups: low education years subgroup (6 or fewer years of education, *n* = 66, group A = 33, group B = 33), medium education years subgroup (7–9°years of education, *n* = 48, group A = 24, group B = 24), and high education years subgroup (10 or more years of education, *n* = 84, group A = 42, group B = 42). The demographic and scale data according to the four groups are presented in Table 1. There were no statistically significant differences in age, sex, MMSE-C, or MoCA-BJ between groups A and B.

**TABLE 1:** Demographic characteristics and cognitive test scores of the samples.

Basic information	Total group subpatients			High education subgroup patients			Medium education subgroup patients			Low education subgroup patients		
	
	A group	B group	*P*-value	A group	B group	*P*-value	A group	B group	*P*-value	A group	B group	*P*-value
Number	161	161		42	42		24	24		33	33	
Age (Mean ± SD)	69.69 ± 11.12	70.01 ± 11.21	0.695	65.83 ± 11.49	67.17 ± 11.76	0.561	64.26 ± 9.07	64.26 ± 9.07	0.397	66.45 ± 9.41	63.97 ± 7.99	0.256
Male	68 (42.2%)	55 (34.2%)	0.136	16 (38.1%)	11 (26.2%)	0.243	8 (33.33%)	8 (33.33%)	0.551	13 (39.4%)	8 (25.00%)	0.215
MMSE-C (Mean ± SD)	12.11 ± 6.82	12.27 ± 6.91	0.761	15.48 ± 6.67	16.17 ± 6.32	0.713	15.54 ± 6.91	15.54 ± 6.91	0.451	11.42 ± 7.08	10.84 ± 5.29	0.901
MoCA-BJ (Mean ± SD)	8.20 ± 5.34	7.91 ± 5.30	0.595	9.62 ± 5.49	10.38 ± 5.70	0.534	8.54 ± 4.92	8.54 ± 4.92	0.563	6.82 ± 5.37	6.00 ± 4.49	0.631

### Applying the circle-arc method for equivalent conversion

Based on the data nadir (x1, y1), zenith (x3, y3), and midpoint (x2, y2) of group A of every subgroup (Table 2), we used the circle-arc formula to calculate the relevant parameters such as xc, yc, and r. The MMSE-C scale in group B was then substituted into the formula to get converted MoCA-BJ of group B. The same flow passes were applied in each subgroup, and the MMSE-C scale and converted MoCA-BJ of group B were shown in Table 3.

**TABLE 2:** Conversion parameter of circle-arc method.

parameters	A group of total group	A group of high education subgroup	A group of medium education subgroup	A group of low education subgroup
(x1, y1)	(2,1)	(2,1)	(2.0)	(3,1)
(x2, y2)	(12,8)	(16,10)	(16,10)	(9,5)
(x3, y3)	(28,25)	(27,20)	(23,16)	(25,15)
(x_c_, y_c_)	(15, 34.75)	(14.5,46.13)	(14, −279.88)	(14, −263.91)
*R*	37.09895	47.79476	280.1321	264.1382

**TABLE 3:** Mini-mental state examination (MMSE-C) and converted montreal cognitive assessment (MoCA-BJ) of group.

MMSE-C	Converted MoCA-BJ of A group of total group	Converted MoCA-BJ of A group of high education subgroup	Converted MoCA-BJ of A group of medium education subgroup	Converted MoCA-BJ of A group of low education subgroup
0	0	0	0	0
1	0	0	0	0
2	1	1	1	0
3	2	2	2	1
4	2	2	2	2
5	3	3	3	2
6	3	3	4	3
7	4	4	4	4
8	5	4	5	4
9	6	5	6	5
10	6	6	6	6
11	7	6	7	6
12	8	7	8	7
13	9	8	8	7
14	10	8	9	8
15	11	9	9	9
16	12	10	10	9
17	13	11	11	10
18	14	12	11	11
19	15	12	12	11
20	16	13	12	12
21	17	14	13	13
22	18	15	14	13
23	19	16	14	14
24	20	17	15	14
25	21	18	15	15
26	22	19	16	16
27	24	20	17	16
28	25	21	17	17
29	26	22	18	17
30	28	23	18	18

### The distribution of mini-mental state examination and original montreal cognitive assessment of group A, converted and original montreal cognitive assessment of group B, and converted montreal cognitive assessment and original mini-mental state examination of group B

**FIGURE 2:**
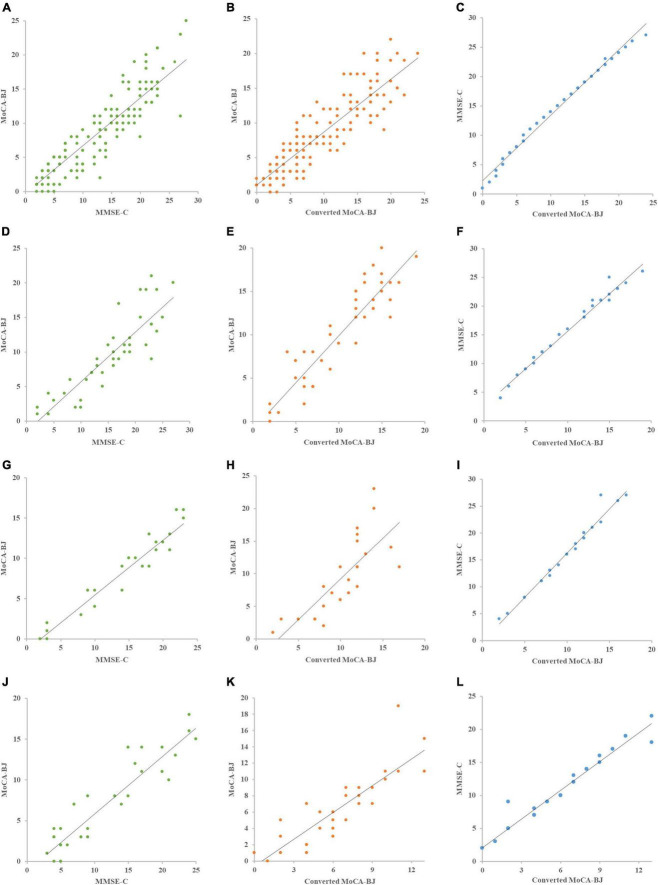
Mini-mental state examination, montreal cognitive assessment, converted MoCA-BJ distribution scatter plot of groups. **(A–C)** The MMSE-C, MoCA-BJ, and converted MoCA-BJ distribution scatter plot of tatol group, **(D–F)** the MMSE-C, MoCA-BJ, and converted MoCA-BJ distribution scatter plot of high education subgroup, **(G-I)** the MMSE-C, MoCA-BJ, and converted MoCA-BJ distribution scatter plot of medium education subgroup, **(J-L)** the MMSE-C, MoCA-BJ, and converted MoCA-BJ distribution scatter plot of low education subgroup.

The original MoCA-BJ and MMSE-C of group A of the total group were approximately linearly distributed, indicating good consistency (Figure 2A). The converted and the original MoCA-BJ in group B of the total group were approximately linear (Figure 2B). The distribution of the converted MoCA-BJ and MMSE-C scores in group B of the total group were also approximately linear (Figure 2C). The original MoCA-BJ and MMSE-C in group A of each subgroup were approximately linearly distributed, indicating good consistency (Figures 2D,G,J). The converted and original MoCA-BJ in group B of each subgroup were approximately linear (Figures 2E,H,K, respectively). The converted MoCA-BJ and MMSE-C in group B of each subgroup were approximately linear (Figures 2F,I,L, respectively).

### Consistency statistical analysis of mini-mental state examination and montreal cognitive assessment of group A and converted montreal cognitive assessment and original montreal cognitive assessment of group B

Consistency analysis of MMSE-C and MoCA-BJ in group A of the total group showed that the correlation coefficient was 0.905 (*P* < 0.001) (Table 4). After equivalent conversion by the circle-arc method, the consistency analysis of the original and the converted MoCA-BJ in group B of the total group showed that the correlation coefficient was 0.891 (*P* < 0.001) (Table 4). The consistency analysis of MMSE-C and MoCA-BJ in group A of each subgroups were 0.874 (*P* < 0.001), 0.949 (*P* < 0.001), and 0.874 (*P* < 0.001) (Table 4), respectively. After equivalent conversion by the circle-arc method, consistency analysis of the original and the converted MoCA-BJ in group B of each subgroups showed that the correlation coefficients were 0.894 (*P* < 0.001), 0.781 (*P* < 0.001), and 0.909 (*P* < 0.001) (Table 4), respectively.

**TABLE 4:** Consistency analysis of montreal cognitive assessment (MoCA-BJ) with mini-mental state examination (MMSE-C) or converted montreal cognitive assessment (MoCA-BJ).

parameters	A group of total group		B group of total group		A group of high education subgroup		B group of high education subgroup		A group of medium education subgroup		B group of medium education subgroup		A group of low education subgroup		B group of low education subgroup	
	
	MMSE-C	MoCA-BJ	MoCA-BJ	Converted MoCA-BJ	MMSE-C	MoCA-BJ	MoCA-BJ	Converted MoCA-BJ	MMSE-C	MoCA-BJ	MoCA-BJ	Converted MoCA-BJ	MMSE-C	MoCA-BJ	MoCA-BJ	Converted MoCA-BJ
Mean	12.11	8.20	7.91	8.98	15.48	9.62	10.38	10.43	15.54	8.54	9.46	10.21	11.42	6.82	6.00	6.06
SD	6.82	5.34	5.30	6.17	6.67	5.49	5.70	4.83	6.91	4.92	5.93	3.73	7.08	5.37	4.49	3.57
*r*	0.905		0.891		0.874		0.894		0.949		0.781		0.874		0.909	
*p*	< 0.001		< 0.001		< 0.001		< 0.001		< 0.001		< 0.001		< 0.001		< 0.001	

## Discussion

In this study, Chinese patients with AD were selected for consistency analysis of MMSE-C and MoCA-BJ scores, and the correlation coefficient was 0.905 (*P* < 0.001), which was consistent with previous findings (Cao et al., 2012; Lam et al., 2013; Chen et al., 2016). In the subgroup stratified by education background, the consistency analysis of the two scales was also statistically significant. This result suggests that the two Chinese versions of the scales are highly correlated and could undergo equivalent conversion.

In this study, MoCA-BJ and MMSE-C were transformed by the circle-arc method and a transformation model was verified. Most previous studies have used the equal percentile equivalence method or the linear equivalence method to convert the MMSE-C and MoCA-BJ scores. For example, the equivalent percentile equivalency method was used in patients with AD (Roalf et al., 2017), all-cause dementia (Bergeron et al., 2017), and Parkinson’s disease (Van Steenoven et al., 2014) to explore the consistency of MoCA-BJ conversion into MMSE-C, and the conclusions were inconsistent. Therefore, later studies have used the equal percentile equivalency method plus linear smoothing to convert MMSE and MoCA in PD and the control group (Lawton et al., 2016), and the results show that the original and the converted MMSE have good consistency, but that makes the calculations harder. The circle-arc method uses the two endpoints and one middle point of the data to obtain the conversion of two kinds of data based on not establishing the computational linear model. The method can be used in small samples (Livingston and Kim, 2009) and can be requires simple calculations. The equivalent design of the arc method can be applied to cases where the available sample of the tester is not enough for equivalent conversion by the traditional method (Sooyeon and Livingston, 2009). The main advantage of the equivalent percentile equivalency method is its accuracy in the upper and lower tail of the score distribution (Sooyeon and Livingston, 2009), which helps avoid the difficulty in equivalent conversion caused by the estimated score probability in the innumerable data area of the scoring scale. In the process of equivalent conversion, the circle-arc method is simple: it involves adding the smoothing model to the equivalent percentile equivalency method.

In this study, a small sample was used to verify the MMSE-C and MoCA-BJ transformation models established by the arc method above. In B group of the total group, the converted MoCA-BJ had a high consistency with the original MoCA-BJ. In addition, subgroup analysis was conducted based on the years of education. No matter if one was in the high years of education, medium years of education, or low years of education subgroups, the converted MoCA-BJ had a high consistency with the original MoCA-BJ in group B, which was consistent with the relevant research results (Van Steenoven et al., 2014; Chen et al., 2016; Helmi et al., 2016; Lawton et al., 2016).

This study had some limitations. First, it was a single-center study and has not been validated in other centers. Second, the conversion of MMSE-C to MOCA-BJ was only performed in patients with AD, and further validation in other patients is required. Although there was stratification according to educational background, the study did not test whether there were differences in conversion between different educational backgrounds. Therefore, the next step is to expand the sample size to verify this transformation.

## Conclusion

This study found that the MMSE-C and MoCA-BJ have higher consistency in Chinese patients with AD. We established and validated a model of MMSE-C and MoCA-BJ score conversion for Chinese patients with AD using the circle-arc method. The transformation model can allow multiple centers and clinical trials to apply the equivalent conversion of MMSE-C scores to MoCA-BJ scores and further apply it to meta-analysis.

## Data availability statement

The original contributions presented in the study are included in the article/supplementary material, further inquiries can be directed to the corresponding author/s.

## Ethics statement

Written informed consent was obtained from the individual(s) for the publication of any potentially identifiable images or data included in this article.

## Author contributions

LW was the guarantor and contributed to the conception of the study. LZ and ZL participated in the analysis and interpretation of the data and drafted the initial manuscript. HL participated in the cognitive assessment. BJ, XL, YZ, and LS revised the article critically for important intellectual content. All authors read and approved the final manuscript.

## References

[B1] Ashford J. W. Borson S. O’Hara R. Dash P. Frank L. Robert P. (2006). Should older adults be screened for dementia? *Alzheimers Dement* 2 76–85. 10.1016/j.jalz.2006.02.005 19595860

[B2] Bergeron D. Flynn K. Verret L. Poulin S. Bouchard R. W. Bocti C. (2017). Multicenter Validation of an MMSE-MoCA Conversion Table. *J. Am. Geriatr. Soc.* 65 1067–1072. 10.1111/jgs.14779 28205215

[B3] Braak H. Braak E. (1996). Evolution of the neuropathology of Alzheimer’s disease. *Acta Neurol. Scand. Suppl.* 165 3–12. 10.1111/j.1600-0404.1996.tb05866.x 8740983

[B4] Cao L. Hai S. Lin X. Shu D. Wang S. Yue J. (2012). Comparison of the saint louis university mental status examination, the mini-mental state examination, and the montreal cognitive assessment in detection of cognitive impairment in chinese elderly from the geriatric department. *J. Am. Med. Dir. Assoc.* 13 626–629. 10.1016/j.jamda.2012.05.003 22698956

[B5] Chen K. L. Xu Y. Chu A. Q. Ding D. Liang X. N. Nasreddine Z. S. (2016). Validation of the chinese version of montreal cognitive assessment basic for screening mild cognitive impairment. *J. Am. Geriatr. Soc.* 64 e285–e290. 10.1111/jgs.14530 27996103

[B6] Crum R. M. Anthony J. C. Bassett S. S. Folstein M. F. (1993). Population-based norms for the Mini-Mental State Examination by age and educational level. *JAMA* 269 2386–2391. 8479064

[B7] Dong Y. Yean Lee W. Hilal S. Saini M. Wong T. Y. Chen C. L. (2013). Comparison of the Montreal Cognitive Assessment and the Mini-Mental State Examination in detecting multi-domain mild cognitive impairment in a Chinese sub-sample drawn from a population-based study. *Int. Psychogeriatr.* 25 1831–1838. 10.1017/S1041610213001129 23870281

[B8] Federico A. Tinazzi M. Tamburin S. (2018). MoCA for cognitive screening in Parkinson’s disease: Beware of floor effect. *Mov. Disord.* 33:499. 10.1002/mds.27329 29460987

[B9] Folstein M. F. Folstein S. E. McHugh P. R. (1975). “Mini-mental state”, A practical method for grading the cognitive state of patients for the clinician. *J. Psychiatr. Res* 123 189–198. 10.1016/0022-3956(75)90026-6 1202204

[B10] Helmi L. Meagher D. O’Mahony E. O’Neill D. Mulligan O. Murthy S. (2016). Agreement and conversion formula between mini-mental state examination and montreal cognitive assessment in an outpatient sample. *World J. Psychiatry* 6 358–364. 10.5498/wjp.v6.i3.358 27679776 PMC5031937

[B11] Huang L. Chen K. L. Lin B. Y. Tang L. Zhao Q. H. Lv Y. R. (2018). Chinese version of Montreal Cognitive Assessment Basic for discrimination among different severities of Alzheimer’s disease. *Neuropsychiatr. Dis. Treat* 14 2133–2140. 10.2147/NDT.S174293 30174426 PMC6110265

[B12] Kasten M. Bruggemann N. Schmidt A. Klein C. (2010). Validity of the MoCA and MMSE in the detection of MCI and dementia in Parkinson disease. *Neurology* 73 1738–1745. 10.1212/WNL.0b013e3181e7948a 20679642

[B13] Lam B. Middleton L. E. Masellis M. Stuss D. T. Harry R. D. Kiss A. (2013). Criterion and convergent validity of the Montreal cognitive assessment with screening and standardized neuropsychological testing. *J. Am. Geriatr. Soc.* 61 2181–2185. 10.1111/jgs.12541 24320735

[B14] Larner A. J. (2012). *Cognitive screening instruments: a practical approach.* London: Springer.

[B15] Lawton M. Kasten M. May M. T. Mollenhauer B. Schaumburg M. Liepelt-Scarfone I. (2016). Validation of conversion between mini-mental state examination and montreal cognitive assessment. *Mov. Disord.* 31 593–596. 10.1002/mds.26498 26861697 PMC4864892

[B16] Lee J. Y. Cho S. J. Na D. L. Kim S. K. Youn J. H. Kwon M. (2008). Brief screening for mild cognitive impairment in elderly outpatient clinic: validation of the Korean version of the Montreal Cognitive Assessment. *J. Geriatr. Psychiatry Neurol.* 21 104–110. 10.1177/0891988708316855 18474719

[B17] Livingston S. A. Kim S. (2009). The Circle-Arc Method for Equating in Small Samples. *J. Educ. Meas.* 46 330–343. 10.1177/0013164419878483 32425217 PMC7221499

[B18] Nasreddine Z. S. Phillips N. A. Bédirian V. Charbonneau S. Whitehead V. Collin I. (2005). The Montreal Cognitive Assessment, MoCA: a brief screening tool for mild cognitive impairment. *J. Am. Geriatr. Soc.* 534 695–699. 10.1111/j.1532-5415.2005.53221.x 15817019

[B19] Nys G. M. van Zandvoort M. J. de Kort P. L. Jansen B. P. Kappelle L. J. de Haan E. H. (2005). Restrictions of the Mini-Mental State Examination in acute stroke. *Arch. Clin. Neuropsychol.* 205 623–629. 10.1016/j.acn.2005.04.001 15939186

[B20] Roalf D. R. Moberg P. J. Xie S. X. Wolk D. A. Moelter S. T. Arnold S. E. (2012). Comparative accuracies of two common screening instruments for classification of Alzheimer’s disease, mild cognitive impairment, and healthy aging. *Alzheimers Dement* 9 529–537. 10.1016/j.jalz.2012.10.001 23260866 PMC4036230

[B21] Roalf D. R. Moore T. M. Mechanic-Hamilton D. Wolk D. A. Arnold S. E. Weintraub D. A. (2017). Bridging cognitive screening tests in neurologic disorders: A crosswalk between the short Montreal Cognitive Assessment and Mini-Mental State Examination. *Alzheimers Dement* 13 947–952. 10.1016/j.jalz.2017.01.015 28238740 PMC5554086

[B22] Siciliano M. Chiorri C. Passaniti C. Sant’Elia V. Trojano L. Santangelo G. (2019). Comparison of alternate and original forms of the Montreal Cognitive Assessment (MoCA): an Italian normative study. *Neurol. Sci.* 40 691–702. 10.1007/s10072-019-3700-7 30637545

[B23] Sooyeon K. Livingston S. A. (2009). Methods of linking with small samples in a common-Item design: An Empirical Comparison. *Educ. Test. Serv.* 2009 i1–i4.

[B24] Sperling R. A. Aisen P. S. Beckett L. A. Bennett D. A. Craft S. Fagan A. M. (2011). Toward defining the preclinical stages of Alzheimer’s disease: recommendations from the National Institute on Aging-Alzheimer’s Association workgroups on diagnostic guidelines for Alzheimer’s disease. *Alzheimers Dement* 73 280–292. 10.1016/j.jalz.2011.03.003 21514248 PMC3220946

[B25] Tombaugh T. N. McIntyre N. J. (1992). The mini-mental state examination: a comprehensive review. *J. Am. Geriatr. Soc.* 409 922–935. 10.1111/j.1532-5415.1992.tb01992.x 1512391

[B26] Tsoi K. K. Chan J. Y. Hirai H. W. Wong S. Y. Kwok T. C. (2015). Cognitive tests to detect dementia: A systematic review and meta-analysis. *JAMA. Intern. Med.* 1759 1450–1458. 10.1001/jamainternmed.2015.2152 26052687

[B27] Van Steenoven I. Aarsland D. Hurtig H. Chen-Plotkin A. Duda J. E. Rick J. (2014). Conversion between mini-mental state examination, montreal cognitive assessment, and dementia rating scale-2 scores in Parkinson’s disease. *Mov. Disord.* 29 1809–1815. 10.1002/mds.26062 25381961 PMC4371590

[B28] Verghese J. Noone M. L. Johnson B. Ambrose A. F. Wang C. Buschke H. (2012). Picture-based memory impairment screen for dementia. *J. Am. Geriatr. Soc.* 60 2116–2120. 10.1111/j.1532-5415.2012.04191.x 23039180 PMC3679906

[B29] Wong G. K. Lam S. W. Wong A. Ngai K. Poon W. S. Mok V. (2013). Comparison of montreal cognitive assessment and mini-mental state examination in evaluating cognitive domain deficit following aneurysmal subarachnoid haemorrhage. *PLoS One* 8:e59946. 10.1371/journal.pone.0059946 23573223 PMC3616097

[B30] Yu J. Li J. Huang X. (2012). The Beijing version of the Montreal Cognitive Assessment as a brief screening tool for mild cognitive impairment: a community-based study. *BMC. Psychiatry.* 12:156. 10.1186/1471-244X-12-156 23009126 PMC3499377

[B31] Zhang M. Y. (2003). *Manual of psychiatric rating scale.* Changsha: Hunan:Hunan Science and Technology Press.

